# Prevention of Atrial Fibrillation After Cardiac Surgery: A Review of Literature and Comparison of Different Treatment Modalities

**DOI:** 10.1097/CRD.0000000000000499

**Published:** 2022-12-20

**Authors:** Jari Halonen, Jussi Kärkkäinen, Helena Jäntti, Tero Martikainen, Antti Valtola, Sten Ellam, Eemu Väliaho, Elmeri Santala, Jenni Räsänen, Auni Juutilainen, Visa Mahlamäki, Sini Vasankari, Tommi Vasankari, Juha Hartikainen

**Affiliations:** From the * Heart Center, Kuopio University Hospital, Kuopio, Finland; † Institute of Clinical Medicine, School of Medicine, University of Eastern Finland, Kuopio, Finland; ‡ Centre for Prehospital Emergency Care, Kuopio University Hospital, Kuopio, Finland; § Department of Anesthesiology and Operative Services, Kuopio University Hospital, Kuopio, Finland; ¶ Department of Clinical Medicine, University of Turku, Turku, Finland; ‖ The UKK Institute for Health Promotion Research, Tampere, Finland; # The Faculty of Medicine and Health Technology, Tampere University, Tampere, Finland.

**Keywords:** atrial fibrillation, postoperative, prevention, cardiac surgery

## Abstract

Atrial fibrillation is the most common arrhythmia to occur after cardiac surgery, with an incidence of 10% to 50%. It is associated with postoperative complications including increased risk of stroke, prolonged hospital stays and increased costs. Despite new insights into the mechanisms of atrial fibrillation, no specific etiologic factor has been identified as the sole perpetrator of the arrhythmia. Current evidence suggests that the pathophysiology of atrial fibrillation in general, as well as after cardiac surgery, is multifactorial. Studies have also shown that new-onset postoperative atrial fibrillation following cardiac surgery is associated with a higher risk of short-term and long-term mortality. Furthermore, it has been demonstrated that prophylactic medical therapy decreases the incidence of postoperative atrial fibrillation after cardiac surgery. Of note, the incidence of postoperative atrial fibrillation has not changed during the last decades despite the numerous preventive strategies and operative techniques proposed, although the perioperative and postoperative care of cardiac patients as such has improved.

New-onset atrial fibrillation (AF) is the most common arrhythmia to occur after cardiac surgery. The reported incidence varies between 5.5% and 57% and is higher after combined coronary artery bypass grafting (CABG) and valve surgery than after CABG alone.^1–4^ AF is especially common after mitral valve surgery, occurring in as many as 64% of patients.^5^ Postoperative AF is the major cause of stroke after on-pump CABG.^6^ According to a recent study, postoperative AF (POAF) was linked with a 4-fold risk of stroke and 3-fold increase of all-cause mortality after CABG.^7^ In addition, it is associated with a need for additional treatment, prolonged hospital stay, and increased costs.^4,8,9^ It is noteworthy that the incidence of POAF has remained unchanged for decades despite rigorous investigation and numerous proposed treatment strategies even with contemporary improvements in cardiac surgical care.^3,10–12^ The new-onset atrial fibrillation starts typically 2 to 3 days postoperatively after cardiac surgery and lasts on average 7 hours.^13^ According to 1 large observational study, women seemed to have a lower risk of POAF, but no effect on the long-term survival between the sexes was found.^14^

Several clinical risk factors for POAF have been recognized, for example, increasing age, male gender, previous history of AF, increased left atrial size, mitral valve disease, previous cardiac surgery, chronic obstructive pulmonary disease (COPD), white race, physical inactivity.^15–17^ Obese patients [body mass index (BMI) *≥*30 kg/m^2^] have a 12% higher risk of POAF compared with nonobese.^18^

Also, perioperative risk factors of POAF have been recognized. Inflammation has a strong relation to the existence of POAF.^19,20^ Other factors include autonomic stimulation, oxidative stress, and atrial stretch related to fluid load.^21,22^ Furthermore, such risk factors as hypokalemia, hypo/hypermagnesemia and increased perioperative ischemia has been shown to be perioperative risk factors for POAF.^23,24^ In addition, there are also some procedural risk factors for POAF: cross-clamp time, location of venous cannulation, perfusion time, prolonged ventilator time, return to the intensive care unit (ICU), and reoperation due to postoperative bleeding have been found to predispose to POAF.^25,26^ Minimal invasive extracorporeal circulation compared with conventional extracorporeal circulation was not useful in the prevention of POAF after CABG.^27^

Some risk score calculators have been developed to help clinicians to identify those patients who are at an increased risk of POAF.^9,28–31^ However, they seem to have a limited ability to predict POAF in cardiac surgical patients.^32^

The effect of beta-blockers, amiodarone, corticosteroids, magnesium, and atrial pacing and left posterior pericardiotomy in the prophylaxis of AF after cardiac surgery has been shown in many studies. In this paper we critically review and compare the effect of different prevention modalities in the prevention of AF, and the impact of prevention on outcome after cardiac surgery.

## BETA-BLOCKERS

The effectiveness of beta-blockers in the prevention of AF after cardiac surgery has been demonstrated in numerous studies. The results of 4 meta-analyses have shown that prophylactic beta-blocker therapy reduces the incidence of AF after cardiac surgery (Table 1). According to the meta-analysis, the type of beta-blocker or the dose have no influence on the effectiveness of the prevention. Yazicioglu and coworkers reported that combining digoxin with atenolol is more effective than atenolol alone.^33^

**TABLE 1.** Clinical Risk Factors of Postoperative Atrial Fibrillation

• Advanced age
• Previous episodes of atrial fibrillation
• Male gender
• Increassed left atrial size
• Mitral valve disease
• White race
• Chronic obstructive pulmonary disease (COPD)
• Physical inactivity
• Previous cardiac surgery
• Obesity (BMI ≥ 30 kg/m^2^)
• Beta-blocker withdrawal
• Renal failure
• Smoking
• Coronary artery disease
• Hypertension

Valtola et al evaluated the bioavailability of perioperative metoprolol tablets in CABG patients in their pharmacokinetics study. Their study showed that the bioavailability of metoprolol is markedly reduced when administered in tablet form during the early phase after CABG.^34^

The study by Balcetyte-Harris et al compared the efficacy and safety of intravenous beta-blocker (esmolol) and oral beta-blocker.^35^ The study was terminated when interim analysis revealed a significantly greater incidence of adverse effects in the group receiving intravenous esmolol, and the lack of any reduction in AF incidence. The efficacy and safety of intravenous propranolol was studied earlier by Abel et al.^36^ They reported that propranolol was more effective in AF prophylaxis than placebo. However, a trend toward more frequent adverse effects in the propranolol treatment group was reported.^36^ Halonen et al randomized 240 patients undergoing CABG to receive oral of intravenous metoprolol with equivalent doses 48 hours after surgery. The incidence of postoperative AF was significantly lower in the intravenous group when compared to the oral group (16.8% vs. 28.1%, *P* = 0.04, respectively). They concluded that intravenous metoprolol is well tolerated and more effective than oral metoprolol in the prevention of AF after cardiac surgery.^37^

In conclusion, the effectiveness of beta-blockers in the prevention of AF after cardiac surgery is confirmed. Indeed, according to the recent guideline, beta-blocking prophylaxis should be given to every patient undergoing cardiac surgery when there are no contraindications for its use.

## SOTALOL

Sotalol is a class III antiarrhythmic agent with beta-receptor and potassium channel blocking properties. These properties theoretically prevent postoperative AF by prolonging refractoriness and blocking neurohormonal activation. The effectiveness of sotalol has been demonstrated in several placebo-controlled trials.^38–40^ The effectiveness of sotalol has also been compared with that of other beta-blockers in 3 randomized trials. In the trial by Parikka and coworkers, sotalol 75 mg/d was compared with metoprolol 120 mg/d in 191 patients who underwent CABG. AF occurred in 32% of the metoprolol group and 16% of the sotalol group. No proarrhythmic effects of sotalol were found during the study.^41^ Similarly, Janssen and coworkers and also Suttorp and coworkers found that sotalol was more effective than metoprolol^42^ or propranolol^43^ in the prevention of AF. Two other studies evaluated the use of sotalol as monotherapy in patients undergoing cardiac surgery.^44,45^ In these studies, sotalol reduced the incidence of postoperative AF by 41% to 93%. A serious limitation in some of the studies was that preoperative beta-blocker therapy was not continued in the control groups, predisposing them to higher rates of AF after cardiac surgery. Forlani et al randomized 207 consecutive CABG patients to receive either magnesium, sotalol, both magnesium, and sotalol or no antiarrhythmic agents. They found that both sotalol and magnesium were effective in reducing the risk of postoperative AF, and that combination therapy of these drugs was the most effective.^46^

A meta-analysis of 8 trials and 1294 patients assessed the effect of sotalol in the prevention of AF after cardiac surgery.^47^ Individual study sample size varied from 36 to 300 patients. The meta-analysis demonstrated that sotalol reduced the incidence of postoperative AF (OR, 0.35; 95% CI, 0.26–0.49) with no significant heterogeneity between trials. Another meta-analysis of 14 trials compared 2583 patients receiving sotalol with 2622 patients receiving either placebo of conventional beta-blockers. Overall, AF was reduced from 33.7% to 16.9% (OR 0.37; 95% CI, 0.29–0.48). On the other hand, significantly more patients were withdrawn from treatment in the sotalol groups than in the placebo groups because of side effects, predominantly hypotension and bradycardia.^48^

Sotalol is potentially a proarrhythmic agent. In nonsurgical patients the proarrhythmic risk has been reported to be 4.3% to 5.9%.^49^ Because of the potential proarrhythmic effects of sotalol, ordinary beta-blockers are considered to be a safer alternative than sotalol in the prevention of AF after surgery.

## AMIODARONE

Amiodarone is a class III antiarrhythmic agent that inhibits multiple ion channels (potassium and calcium) and adrenergic receptors (α and β). Amiodarone has been shown to be useful in the prevention of postoperative AF. Studies in which amiodarone was given orally starting 1 or several days preoperatively report that the incidence of AF decreased by 41% to 53% and the length of AF shortened when compared with placebo.^50–53^

The effect of intravenous amiodarone therapy has also been evaluated. In these studies, the amount of amiodarone given has varied between studies. Amiodarone has reduced the incidence of AF by 25% to 76% compared with placebo.^54–56^

A few studies have not found amiodarone to be effective in the prevention of AF after cardiac surgery. However, the number of patients has been small in these studies, and they are underpowered to draw any conclusion.^57–59^

A meta-analysis of 9 randomized trials showed that amiodarone therapy decreased the incidence of AF from 37% to 22.5%.^47^ However, amiodarone was not found to be a cost-effective alternative for all patients undergoing CABG. In contrast, elderly patients, patients with COPD and patients undergoing both bypass and valvular surgery possibly benefit from amiodarone.^60^ Other meta-analyses have also examined the feasibility of amiodarone in the prevention of AF after cardiac surgery.^48,61–63^ Aasbo et al combined the data of 10 trials and reported a significant reduction in the incidence of AF or flutter (RR 0.64; 95% CI, 0.21–0.76) with amiodarone therapy versus placebo.^61^ Studies of oral and intravenous routes of amiodarone administration yielded similar results. The length of hospital stay was also significantly reduced with amiodarone. Gillespie et al reported in their meta-analysis of 15 trials a 50% reduction in postoperative AF with amiodarone treated patients versus placebo treated patients.^62^ The type of surgery, use of beta-blockers, and route of the amiodarone administration did not have significant effects on the overall results of the analysis.

The safety of amiodarone was evaluated in a meta-analysis of 18 randomized controlled trials and 3408 patients.^63^ The authors reported that the use of amiodarone was associated with increased risk of bradycardia and hypotension, although the risk of heart block, nausea, and myocardial infarction was not significantly increased. The rates of bradycardia and hypotension were higher in studies using intravenous amiodarone than in those using oral amiodarone.

Amiodarone cannot be recommended to be given routinely to all patients undergoing heart surgery. On the other hand, according to the guideline’s amiodarone therapy can be considered for patients who are at an especially high risk (old patients, previous episodes of AF, valve surgery) for developing AF postoperatively.

## MAGNESIUM

Low serum magnesium level is common after cardiac surgery.^64,65^ Low magnesium concentration is also an independent determinant of AF after CABG.^66^ Moreover, this association has been noted even when serum magnesium concentrations do not correlate with intracellular or myocardial magnesium concentrations.^67^

Administration of intravenous magnesium has been shown to decrease the incidence of AF after cardiac surgery.^64,68–72^ In a randomized study in which patients received either magnesium 178 mEq or placebo for 4 days following surgery, the incidence of AF was lower in the magnesium group than in the placebo group.^64^ Wistbacka and coworkers evaluated the role of magnesium dosage in the prevention of AF. In the high dose magnesium group (4.2 g before surgery, 11.9 g infusion by the morning of the first postoperative day and 5.5 g on the following day), the incidence of AF was lower than in the low dose group (4.2 g, 2.9 g, 1.4 g). Magnesium concentration was also normal in patients receiving the low dose magnesium.^69^ In a trial in which 200 CABG patients were randomized to receive either 6 mmol/days of magnesium or placebo on the day before surgery and the first 4 days after surgery, the incidence of AF was only 2% in the magnesium group, but 21% in the placebo group.^70^ In contrast, Jensen and coworkers found that magnesium decreased the duration of AF and flutter but did not decrease the incidence of AF.^72^ In a retrospective study patient undergoing off-pump CABG who received magnesium were less likely to experience postoperative AF than control patients (12% vs. 29%, respectively).^73^

Negative studies about the preventive effect of magnesium on postoperative AF have also been published. In a study by Parikka, 70 mmol of magnesium was given during the first 48 hours after surgery. Magnesium did not reduce the incidence of AF, and surprisingly a high serum magnesium level was found to increase the incidence of AF.^74^ Another study in which 14.4 g of magnesium was given during the first 24 hours postoperatively found no effect of magnesium on the incidence of supraventricular tachycardias.^75^

In a meta-analysis of 20 studies and 2490 patients, magnesium decreased the incidence of POAF from 28% to 18%.^76^ The effectiveness of magnesium has been shown also in other meta-analyses.^77,78^

In conclusion, it seems that magnesium reduces the risk of AF after cardiac surgery. The optimum dose remains to be determined. There is no evidence that magnesium would be of benefit for patients who already are on beta-blocker medication.

## CORTICOSTEROIDS

Cardiac surgery with extracorporeal circulation is known to be associated with systemic inflammatory response,^79,80^ which may be in part responsible for postoperative AF. Complement, C-reactive protein complex level, and number of white blood cells (markers of inflammatory reaction) are increased in patients who develop AF.^81,82^ Corticosteroids have anti-inflammatory activity and reduce exaggerated inflammatory reaction.^83^ Prospective randomized trials in nonoperative patients have reported that corticosteroid therapy reduces the risk of recurrent and permanent AF in patients converted from their first episode of AF.^84^

A double-blind, randomized multicenter trial assessed the the efficacy of corticosteroid in the prevention of AF after cardiac surgery.^85^ Two hundred forty-one patients scheduled to undergo cardiac surgery were randomized to receive either 100 mg intravenous hydrocortisone or placebo 1 dose every 8 hours during the 3 postoperative days. The incidence of postoperative AF was significantly lower in the hydrocortisone group than in placebo group (30% vs. 48% respectively, adjusted hazard ratio 0.54, *P* = 0.004). There were no complications related to the use of hydrocortisone.

The effects of corticosteroid treatment on postoperative AF have been addressed also in 2 randomized controlled trials with postoperative AF as the primary end point. The study by Prasongsukarn et al studied 86 patients scheduled for CABG surgery who were administered 1000 mg of methylprednisolone or placebo before surgery and 4 mg of dexamethasone or placebo every 6 hours for 24 hours after surgery. The postoperative incidence of AF was significantly lower in the corticosteroid group than in the placebo group (21% vs. 51%, respectively).^86^ Halvorsen et al administered 4 mg of dexamethasone or placebo after induction of anesthesia and on the first postoperative morning in 300 patients undergoing CABG surgery. They failed to demonstrate the reducing effect of corticosteroid on the incidence of postoperative AF.^87^

Two other studies deserve to be mentioned. Rubens et al enrolled 68 patients undergoing CABG and randomized them to 1000 mg intravenous infusion of methylprednisolone or placebo before the surgery. Methylprednisolone was found to have a statistically significant inhibitory effect on the incidence of postoperative AF (12% in the treatment group vs. 34% in the placebo group).^88^ Yared et al studied 235 patients scheduled for CABG or valve surgery. The patients were given a single dose of 0.6 mg/kg of dexamethasone or placebo after induction of anesthesia. Compared with the placebo group, the dexamethasone group had a lower incidence of postoperative AF (19% vs. 32%, respectively).^89^ However, in these trials, postoperative AF was not a primary end point, and they had no prospective definition of AF. Thus, these studies were not primarily designed to address the effect of corticosteroids on postoperative AF, but on the activation of inflammatory and coagulation pathways and recovery from cardiac surgery.

A meta-analysis analyzed the effect of perioperative corticosteroid use on the incidence of AF after cardiac surgery. Nine studies with 990 patients were included in the meta-analysis, and it was found that corticosteroids significantly lowered patients’ odds of developing postoperative AF by 45% (OR 0.55; 95% CI, 0.39–0.78).^90^ Another meta-analysis of 50 randomized controlled trials and 3323 patients reported that corticosteroid prophylaxis reduced the risk of AF (RR 0.74; 95% CI, 0.63–0.86; *P* < 0.01).^91^ A third meta-analysis consisting of 14 studies and 13,803 patients, showed that confirmed that steroids reduced new-onset AF (RR 0.70; 95% CI, 0.55–0.89).^92^ We conclude that there is solid evidence to show that corticosteroid treatment decreases the risk of AF after CABG and valve surgery. We conclude that there is solid evidence to show that corticosteroid treatment decreases the risk of AF after cardiac surgery after CABG and valve surgery.

## STATINS

A few studies have reported the use of statins in relation to the development of postoperative AF. The main limitation of these statin studies is that many of them are nonrandomized and based on different kind of registry analyses. Numerous mechanisms have been proposed to explain a possible protective effect of statins in the prevention of postoperative AF after cardiac surgery including antioxidant effects, direct antiarrhythmic effects mediated through cell membrane stabilization, protection of ischemic myocardium, and anti-inflammatory effects.^2,93–96^ Although the precise mechanisms by which statins may prevent AF have not yet been identified, it is likely that the effects are multifactorial.

The ARMYDA-3 (Atorvastatin for Reduction of Myocardial Dysrhythmia after cardiac surgery) was the first randomized and placebo-controlled trial on statin, it showed a significant decrease in AF incidence in atorvastatin treated patients. Postoperative AF occurred in 35 (35%) of 101 patients in the atorvastatin group versus 56 (57%) of 99 patients in the placebo group. Also, the postoperative hospital stay was significantly lower in the atorvastatin group compared with placebo group (6.3 ± 1.2 vs 6.9 ± 1.4 days).^2^

In an observational study of 362 patients (267 on and 95 not on statin medication) the postoperative AF was less frequent, and its duration was shorter in the statin group compared to the non–statin group (8.2% vs. 16.8%, respectively). One important limitation of this study was that the recognition of AF episodes was not based on continuously ECG recording.^97^ In another study of 234 patients undergoing CABG, 28.2% experienced postoperative AF. Multivariate analysis found that the risk of postoperative AF was decreased with statin use (OR 0.52; 95% CI, 0.28–0.96; *P* < 0.01). However, there were some noteworthy limitations of this study; it was retrospective, included patients with previous history of AF and various statin regiments were used and also the study population was too small to conclude the exact effect of statins in the prevention of AF after cardiac surgery.^93^ A retrospective analysis on statins and postoperative AF among patients receiving amiodarone and beta-blocker therapy prophylactically showed that adjunctive statin pre–treatment decreased postoperative AF by 40%.^98^ Mariscalco et al studied 405 patients undergoing isolated CABG procedures. Postoperative AF occurred in 29.5% of the patients with preoperative statin therapy compared with 40.9% of those patients without statin use (*P* = 0.017). Overall, preoperative statin use was associated with a 42% reduction in the risk of postoperative AF.^99^

Nonetheless, there are also negative studies showing minimal or no benefit of statins in the prevention of postoperative AF.^100^ Virani et al studied a total of 2096 patients undergoing cardiac surgery, including isolated valve surgery and patients with low ejection fraction. AF occurred in 31.4% in both the statin and nonstatin groups. However, this study was retrospective, patients having received different doses of several statins, and also the data concerning duration of statin use prior to cardiac surgery were incomplete.^100^ We conclude that the role of statins in the prevention of postoperative AF after cardiac surgery is not fully established.

## OTHER MEDICAL THERAPY

Digoxin has not been found effective in the prevention of postoperative AF.^101–103^ Verapamil also seems to be ineffective.^101^ Intravenous diltiazem has been compared with intravenous nitrate in the prevention of AF in 4 different studies.^104–107^ In these studies, diltiazem turned out to be more effective than nitrates. On the other hand, no placebo-controlled trials with diltiazem have been carried out. According to a meta-analysis by Wijeysundera et al, diltiazem has no significant effect on supraventricular tachycardias (SVT) after cardiac surgery (OR 0.73; 95% CI, 0.48–1.12). A subgroup analysis in a meta-analysis of calcium channel blockers found that nondihydropyridines significantly suppressed postoperative supraventricular arrhythmias (OR 0.62; 95% CI, 0.41–0.93), but with a high heterogeneity.^108^ In 1 randomized controlled trial, triiodothyronine was found to decrease postoperative AF in patients who had a low left ventricular ejection fraction.^109^

Anglade et al demonstrated that the preoperative use of thiazolidinedione, which has some anti-inflammatory properties in diabetic patients undergoing cardiac surgery, was associated with a 20% reduction in postoperative AF, but it did not reach statistical significance due to the study being underpowered.^110^

A large prospective observational study of 4657 patients undergoing cardiac surgery demonstrated that postoperative use of angiotensin-converting enzyme inhibitor (ACEI) reduced the incidence of postoperative AF after cardiac surgery by 38%.^111^ Negative studies about the preventive effect of ACEIs and angiotensin receptor blockers (ARB) on postoperative AF have also been published. Coleman et al performed a single center, retrospective study of 1469 matched patients undergoing cardiac surgery. They found that postoperative ACE inhibitor use was not associated with a reduction of postoperative AF after cardiac surgery.^112^ Similarly, data extracted from 2 randomized controlled trials (AFIST II and AFIST III) showed that preoperative use of ACE inhibitors or ARB was not associated with a significant reduction in postoperative AF.^113^

In a prospective trial with 160 patients, the efficacy of preoperative and postoperative treatment of n-3 polyunsaturated fatty acid (PUFA) were assessed. The incidence of postoperative AF was lower in the PUFA group than in the control group 15% versus 33%, respectively.^114^ The effect of a new antiarrhythmic agent, dronedarone, has not been assessed in the prevention of AF after cardiac surgery. The efficacy of omega-3 fatty acids for the prevention of recurrent AF has been studied in nonoperative patients, but not in the prevention of AF after cardiac surgery.^115^

## ATRIAL PACING

The effectiveness of atrial pacing in the prevention of AF occurring after cardiac surgery has been investigated in many studies. Gerstenfeld et al demonstrated that biatrial and right atrial pacing were well tolerated and safe.^116^ In another study, they found that biatrial pacing combined with beta-blockers decreased the incidence of postoperative AF. Patients older than 70 years especially seemed to benefit from the combination treatment.^117^ In a randomized trial of 154 patients, dynamic right, left and biatrial pacing decreased the incidence of AF compared with the control group.^118^ Correspondingly, in another study dynamic right atrial overpacking decreased postoperative AF after cardiac surgery.^119^ In a study comparing the location of atrial pacing, biatrial pacing was more effective than right or left atrial pacing.^120^ The effectiveness of biatrial pacing in the prevention of postoperative AF has also been reported by Levy et al.^121^ In their prospective study with 118 patients, biatrial pacing but not right or left atrial pacing decreased the incidence of postoperative AF.^122^ In a randomized trial of 100 patients, AAI pacing (pacing cutoff 10 beats above the normal pulse rate) were compared with no pacing. No difference was found between the groups. Instead, more atrial premature contractions were found in the pacing group.^123^ In another study by Kurz et al, biatrial pacing was compared with pharmacological treatment in the prevention of AF. The trial was terminated early because of proarrhythmias in the pacing group.^124^ In a study by Hakala et al, dynamic right atrial overpacing or prevention of bradycardia did not decrease the incidence of postoperative AF.^125^

Two meta-analyses have investigated the effect of pacing on AF incidence after cardiac surgery. In a meta-analysis by Crystal et al, 10 studies and 1473 patients, 3 different methods of atrial pacing (right, left, and biatrial) were compared. Only biatrial pacing was shown to decrease the incidence of postoperative atrial AF.^47^ Similarly, another meta-analysis by Burgess et al showed that only biatrial pacing had a significant effect in reducing the incidence of AF from an average of 35.3% in the control group to 17.7% in the paced group.^48^ In conclusion, it seems that biatrial pacing has beneficial effects on reducing the risk of postoperative AF after cardiac surgery. Several pacing protocols have been used in the prophylaxis of POAF. Biatrial pacing has been shown to be the most efficient pacing protocol, especially combined with beta-blockers.^48,116,118^ In the prospective, randomized open trial,^126^ 165 consecutive CABG patients were randomized to receive either intravenous beta-blocker metoprolol or biatrial overdrive pacing and oral metoprolol (50–150 mg daily) for 72 hours after CABG starting immediately after surgery. The incidence of POAF did not differ from each other (14% vs. 18%, respectively, *P* = 0.66).

## POSTERIOR PERICARDIOTOMY

The concept of opening the posterior pericardium to prevent AF is based on the assumption that this would decrease the accumulation of pericardial fluid postoperatively. The posterior pericardium is usually opened with a 4 cm longitudinal incision. Mulay et al were the first to report the effectiveness of this procedure in the prevention of postoperative AF.^127^ They found that the occurrence of a significant accumulation of pericardial fluid on echocardiogram decreased from 40% in the control group to 8% in the intervention group. At the same time, the incidence of supraventricular tachyarrhythmias decreased from 36% to 8%. Two other randomized trials have also found that posterior pericardiotomy decreases the incidence of postoperative AF.^128,129^

In contrast, in a prospective, controlled trial of 100 patients, posterior pericardiotomy had no effect on POAF.^130^ In the recent published prospective randomized study of 420 patients undergoing elective CABG, aortic valve surgery, ascending aorta, or a combination of these were included and randomly assigned to the left posterior pericardiotomy group (n = 212) or the no intervention group (n = 208). The incidence of POAF was significantly lower in the posterior left pericardiotomy group than in the no intervention group, [37 (17%) of 212 vs. 66 (32.0 %) of 208, p= 0.007].^131^

## OTHER POTENTIALLY USEFUL STRATEGIES

Oxidative stress has been suggested to have a role in the pathogenesis of AF, and some trials have tested antioxidative agents for the prevention of postoperative AF after cardiac surgery. Ozaydin et al studied in their prospective, randomized, double blind, and placebo-controlled study with 115 patients the effect of N-Acetylcysteine on postoperative AF. The incidence of AF was significantly lower in the treatment group than in the placebo group.^132^

In another study, Carnes et al tested the hypothesis that perioperative oxidative stress has a significant role in the etiology of AF. Of the patients treated with ascorbate, 16.3% developed AF or flutter, compared with 34.9% in the control group, the difference being statistically significant.^133^

The American College of Chest Physicians guidelines also recommend mild hypothermia, the use of posterior pericardiotomy and heparin coated CPB circuits as possible intraoperative preventive strategies for the reduction of AF following cardiac surgery.^134^

The efficacy and safety of botulinum toxin injections into epicardial fat pads to reduce AF inducibility has been evaluated in a a randomized, double-blind study population of 60 patients.^135^ Postoperative AF occurred in 7% of the study group and 30% in the placebo group with log rank test p-value of 0.024. The lack of a mechanistic explanation was regarded as the major study limitation.

Vitamin D deficiency has been observed to increase the prevalence of POAF,^136^ and therapy with vitamin D has been experimented to prevent POAF.^137^ Talasaz et al performed a prospective, open-label, randomized clinical trial in which 196 vitamin D deficient [25(OH)D level < 30 ng/mL] patients were included to receive either standard care or standard care and vitamin D3 600 000 IU 5 days before surgery. The incidence of POAF, as well as the length of ICU stay, was significantly lower in the treatment versus standard care group (9.68% vs. 20.39%, *P* = 0.038).^138^

As colchicine monotherapy has been suggested to prevent POAF,^139^ COPPS-2 trial (investigator-initiated, double-blind, placebo-controlled, and randomized trial including 360 candidates for cardiac surgery) was conducted to determine the efficacy and safety of perioperative administration of oral colchicine starting between 48 and 72 hours before surgery and continuing for 1 month after surgery to reduce postpericardiotomy syndrome, POAF, and postoperative pleural and pericardial effusions. Patients receiving colchicine treatment had more adverse effects compared to placebo, mainly due to gastrointestinal intolerance, leading to discontinuation in 20% of study subjects. POAF occurred in 33.9% in the colchicine group and in 41.7% in the placebo group, with absolute difference of 7.8% (95% CI, –2.2%–17.6%). However, the respective results from the prespecified on-treatment analysis differed between the treatment groups, with POAF incidence of 27.9% in the colchicine group versus 41.2% in the placebo group with an absolute difference of 14.2% (95% CI, 3.3%–24.7%). Although colchicine therapy did not reduce the incidence of POAF, it did reduce the incidence of postpericardiotomy syndrome.^140^

Clinical efficacy of epicardial application of drug-releasing hydrogels has been tested with the aim to increase drug efficacy and reduce side effects. To reduce POAF, amiodarone hydrogel was compared to corticosteroid hydrogel in a randomized prospective study with 150 CABG patients.^141^ Both amiodarone and triamcinolone hydrogels were sprayed diffusely over the biatrial epicardium, while the control group underwent the procedure with hydrogel spray only. The plasma amiodarone and triamcinolone concentrations in the atria were significantly higher than in extracardiac tissues, but plasma concentrations remained below the detection limits. The incidence of POAF was 8% in amiodarone hydrogel and 22% in the triamcinolone hydrogel group (*P* < 0.01).

Thus far, the utility of the aforementioned potential treatments and approaches remains unclear, with verification demanding future research.

## COMPARISON OF DIFFERENT PREVENTION MODALITIES

A number of studies of the prevention of AF after heart surgery have been published. However, not many of them have compared different prophylaxis methods. The efficacy of metoprolol and amiodarone was compared in randomized, prospective, equivalence, multicenter study. Authors randomized 316 patients undergoing cardiac surgery to receive either intravenous metoprolol or intravenous amiodarone for 48 hours after the surgery. The occurrence of AF was similar in the metoprolol and amiodarone group. However, because of the wide range of the confidence interval, the authors cannot conclude that the 2 treatment arms were equally effective.^142^

In a prospective, randomized, double-blind and placebo-controlled study, 253 patients were randomized to receive orally administered amiodarone and metoprolol, only metoprolol and only sotalol or only placebo. In patients receiving the combination medication (amiodarone + metoprolol) and patients receiving sotalol, the occurrence of AF decreased from 53.8% in the placebo group to 30.2% and 31.7%, whereas in the metoprolol group its occurrence was 40.3%. Treatment effects did not differ significantly between the active drug groups.^45^

Cardona et al, in turn, compared right atrial pacing, intravenously administered amiodarone, and orally administered beta-blocker medication with regard to preventing AF after heart surgery and the duration of hospital stay. However, the sample size was too small for any conclusions to be drawn from their results.^143^

Wurdeman et al compared in their meta-analysis the effectiveness of amiodarone and sotalol in the prevention of AF after cardiac surgery. Ten randomized and controlled trials with 1303 patients were included in the meta-analysis. Both amiodarone and sotalol were more effective than placebo treatment in the prevention postoperative AF. There were no differences between amiodarone and sotalol.^144^

In their meta-analysis Zimmer et al studied 13 trials and 1783 patients. In this meta-analysis the efficacy of amiodarone, sotalol, pacing and amiodarone were compared. They did not find any difference between these treatment modalities as regards the efficacy of AF prophylaxis.^145^

In another meta-analysis by Crystal et al, 52 randomized trials of beta-blockers, sotalol, amiodarone, or pacing were studied in the prevention of AF after cardiac surgery. Each of the 3 drug treatments were effective in the prevention of AF, with the following odds ratios: sotalol, 0.35; beta-blockers, 0.39; and amiodarone, 0.48. Pacing was also effective: for biatrial pacing the OR was 0.46. They concluded that beta-blockers, amiodarone and sotalol all reduced the risk of postoperative AF after cardiac surgery, with no marked difference between them.^47^

In their meta-analysis Burgess et al analyzed 94 trials using beta-blockers, sotalol, amiodarone, magnesium, overdrive pacing, digoxin, and calcium channel blockers in the prevention of AF after cardiac surgery. Amiodarone, sotalol, beta-blockers, magnesium and atrial pacing were effective in the prevention of AF after cardiac surgery. However, they concluded that the effect of beta-blockers is less than previously thought.^48^

## IMPACT OF PREVENTION ON OUTCOME AFTER CARDIAC SURGERY

Beta-blockers, sotalol, amiodarone, biatrial pacing and magnesium are effective in the prevention of AF after cardiac surgery. In a meta-analysis of 52 prospective randomized trials, the preventive strategy of AF reduced the length of hospital stay by somewhat less than half a day: conventional beta-blockers or sotalol did not significantly reduce the length of hospital stay amiodarone reduced the length of hospital stay significantly by 0.91 days. The incidence of stroke was not reduced by AF prophylaxis in this meta-analysis.^47^ Another meta-analysis comparing AF prophylaxis (amiodarone, sotalol, pacing, and procainamide) with placebo involved 13 trials and 1783 patients. The results revealed a decrease in the length of hospital stay by mean of 1.0 days with the various modalities of antiarrhythmic prophylaxis. However, there was no significant reduction in the cost of the treatment. In addition, the maintenance of sinus rhythm did not decrease the incidence of stroke.^145^

Meta-analysis of 49 prospective randomized trials assessed the efficacy of conventional beta-blockers, amiodarone, sotalol, magnesium, and atrial pacing in the prevention of AF after cardiac surgery. Only amiodarone and atrial pacing significantly reduced the length of hospital stay (–0.60 days, respectively). Collectively, all treatments analyzed together also reduced the incidence of stroke (OR 0.6). Amiodarone was the only intervention that alone reduced the risk of stroke (OR 0.54).^48^

## QUESTIONS WHICH REMAIN TO BE ANSWERED

Although the prevention of postoperative AF has been excessively studied some questions still remain to be answered. The optimum dose of magnesium in the prevention of AF is unclear. Neither we know if there any benefit of magnesium in the prevention of AF when patients are treated with beta-blockers. There is not enough data comparing the different treatment modalities. For example, the role of atrial pacing in the prevention of AF is unclear. It is not known if it offers any additional value in the AF prophylaxis, on top of corticosteroids or intravenous beta-blockers.
